# Genome-wide analyses of lung cancer after single high-dose radiation at five time points (2, 6, 12, 24, and 48 h)

**DOI:** 10.3389/fgene.2023.1126236

**Published:** 2023-03-03

**Authors:** Yajing Du, Yunna Zheng, Kaiwen Yu, Cheng Zhan, Tiankui Qiao

**Affiliations:** ^1^ Center for Tumor Diagnosis and Therapy, Jinshan Hospital, Fudan University, Shanghai, China; ^2^ OE Biotech Co., Ltd., Shanghai, China; ^3^ Shanghai Chest Hospital, Shanghai Jiaotong University, Shanghai, China; ^4^ Department of Thoracic Surgery, Zhongshan Hospital, Fudan University, Shanghai, China

**Keywords:** whole-transcriptome sequencing, non-small cell lung cancer, radiobiology, precision radiotherapy, bioinformatics

## Abstract

**Background:** An increasing number of clinicians are experimenting with high-dose radiation. This study focuses on the genomic effects of high-dose single-shot radiotherapy and aims to provide a dynamic map for non-small cell lung cancer (NSCLC).

**Methods:** We used whole-transcriptome sequencing to understand the evolution at molecular levels in A549 and H1299 exposed to 10 Gy X-rays at different times (2, 6, 12, 24, and 48 h) in comparison with the no radiation group. Ingenuity pathway analysis, ceRNA analysis, enrichment analysis, and cell cycle experiments are performed for molecular analyses and function analyses.

**Results:** Whole-transcriptome sequencing of NSCLC showed a significant dynamic change after radiotherapy within 48 h. MiR-219-1-3p and miR-221-3p, miR-503-5p, hsa-miR-455-5p, hsa-miR-29-3p, and hsa-miR-339-5p were in the core of the ceRNA related to time change. GO and KEGG analyses of the top 30 mRNA included DNA repair, autophagy, apoptosis, and ferroptosis pathways. Regulation of the cell cycle-related transcription factor E2F1 might have a key role in the early stage of radiotherapy (2.6 h) and in the later stage of autophagy (24 and 48 h). Functions involving different genes/proteins over multiple periods implied a dose of 10 Gy was related to the kidney and liver pathway. Radiation-induced cell cycle arrest at the G2/M phase was evident at 24 h. We also observed the increased expression of CCNB1 at 24 h in PCR and WB experiments.

**Conclusion:** Our transcriptomic and experimental analyses showed a dynamic change after radiation therapy in 48 h and highlighted the key molecules and pathways in NSCLC after high-dose single-shot radiotherapy.

## 1 Introduction

Lung cancer (LC) accounted for the world’s highest mortality rate and second-highest incidence rate in 2022 (Siegel et al., 2022). Radiotherapy (RT) can cure about 40% of cancers (De Ruysscher et al., 2019), which has bright therapeutic prospects for patients.

Precision radiotherapy aims to optimize outcomes and minimize toxicity to patients (Joseph and Vijayakumar, 2020). Most researchers are currently studying the balance of the dose (Scott et al., 2021) or the spatial depth per fraction to decrease side effects. By using artificial intelligence, dose distributions can be predicted based on the anatomy of a patient and calculated more quickly (Hosny et al., 2018; Huynh et al., 2020; Luk et al., 2022; Teuwen et al., 2022). In clinical practice, doctors usually adapt 24 h or 48 h/fraction (fx). For a high dose (such as 10–12 Gy), the total time of five fractions can range from 1.5 to 2 weeks (Chmura et al., 2021).The hours of the fraction are not accurate, and few studies discussed suitable hour of fraction, involving less dynamic changes.

In recent years, precision radiotherapy applied high-dose therapy (Burkoň et al., 2022; Chairmadurai et al., 2022; DLP et al., 2022; Milic et al., 2022; Sidaway, 2022; Tadimalla et al., 2022). Stereotactic body radiotherapy (SBRT) has the characteristics of high tumor dose distribution in the irradiation center and a rapid drop of extradural dose. For lung cancers that are early-stage and inoperable, this is the standard radiation therapy (Lo et al., 2010; Timmerman et al., 2014). The efficacy and toxicity of stereotactic body radiotherapy in patients with centrally located non-small cell lung cancer (10–12 Gy/fraction) were studied (Chmura et al., 2021).

Our study designed groups after radiation for 2, 6, 12, 24, and 48 h to reveal the characteristics of different time periods to discover the suitable interval time for multi-fractions and explore the dynamic change of a gene caused by radiation in single-fraction therapy. Meanwhile, we used the whole-transcriptome sequencing method to learn radiobiology from the perspective of a genome. This enriches the radiobiological content of high-dose radiation therapy, providing biological basics for treatment of SBRT and suggesting new possible molecular methods for combined targeted therapy and chemotherapy.

## 2 Materials and methods

### 2.1 Ethics statement

The Ethics Committees of Jinshan Hospital of Fudan University exempted the study because no personal information is included in the study.

### 2.2 Transcriptome sequencing sample preparation

For the present study, NSCLC cells (A549 and H1299) in a six-well plate at 40% density were divided into no radiation and radiation groups. The radiation group was split into five time points, with two repeats per group.

The radiation group was exposed to a single high dose (Trilogy linear accelerator, 6 MV X-ray radiation, absorption dose rate of 600 cGy/min, once, 10 Gy dose). The cells were washed with PBS twice. TRIzol was added to lysis cells at 2, 6, 12, 24, and 48 h after radiation with the no radiation group. The whole transcriptome was sequenced in a total of 24 samples.

### 2.3 Cell cycle assays

A549 and H1299 were collected at 2, 6, 12, 24, and 48 h after radiation and fixed in 70% ethyl alcohol at −20°C overnight together with the no radiation group. For 15 min, they were incubated in 0.5 mL PI/RNase Staining Buffer (BD Biosciences, Franklin, NJ, United States) after three washes with PBS. Flow cytometry was used to analyze the cell fractions (Beckman Coulter or BD Biosciences in the United States).

### 2.4 Western blot detection

Cells were washed twice with PBS and lysed at 4°C for 30 min. Purities were selected by centrifugation at 15,000*g, at 4°C for 20 min, 10% SDS-PAGE was used to separate proteins, and a nitrocellulose filter was used for transfer. All samples were evenly transferred and incubated in a closed solution for 2 hours at room temperature using a stained filter. Anti-CCNB1 was diluted at 1:1000 for 12 h, washed twice with PBS and TBST, and then exposed to the filter. The filter was incubated with the secondary antibody, at 1:1000 for 1 h, and then washed with TBST. In addition, anti-β-actin antibodies were used as an internal reference.

### 2.5 Real-time fluorescence quantitative polymerase chain reaction detection

The RNA Purification Kit (Yishan Biotechnology Company, Shanghai, China) and the 5x Reverse Transcriptase Master Mix (Takara, Osaka, Japan) were used to obtain cDNA. The primers were as follows: β-actin, forward 5′-TGA​CGT​GGA​CAT​CCG​CAA​AG-3′, reverse 5′-CTG​GAA​GGT​GGA​CAG​CGA​GG-3′; CCNB1, forward 5′-AAT​AAG​GCG​AAG​ATC​AAC​ATG​GC-3′, reverse 5′-TTT​GTT​ACC​AAT​GTC​CCC​AAG​AG-3′.

### 2.6 Bioinformatics analysis

#### 2.6.1 Differential mRNA, miRNA, circRNA, and lncRNA

The original data were standardized. The mRNA, miRNA, circRNA, and lncRNA of the 2-, 6-, 12-, 24-, and 48-h treatment groups and the no radiation group were analyzed by the DESeq package of the R language software. |log2 (fold change) | >1 and *p* < 0.05 were set as the criteria for intergroup differences.

The no radiation group of A549 and H1299 was also analyzed with the DESeq package to obtain the differential mRNA, miRNA, circRNA, and lncRNA, named as NCdiffmRNA, NCdiffmiRNA, NCdiffcircRNA, and NCdifflncRNA, respectively. |log2 (fold change) | >1 and *p* < 0.05 were set as the criteria. These NCdiffRNAs (NCdiffmRNA, NCdiffmiRNA, NCdiffcircRNA, and NCdifflncRNA) represent the difference caused by the cell line. A549 is an epithelial cell isolated from the lungs of a 58-year-old white male with carcinoma. H1299 is isolated from the lungs of a 43-year-old white male patient with carcinoma.

#### 2.6.2 Short Time-series Expression Miner

**FIGURE 1:**
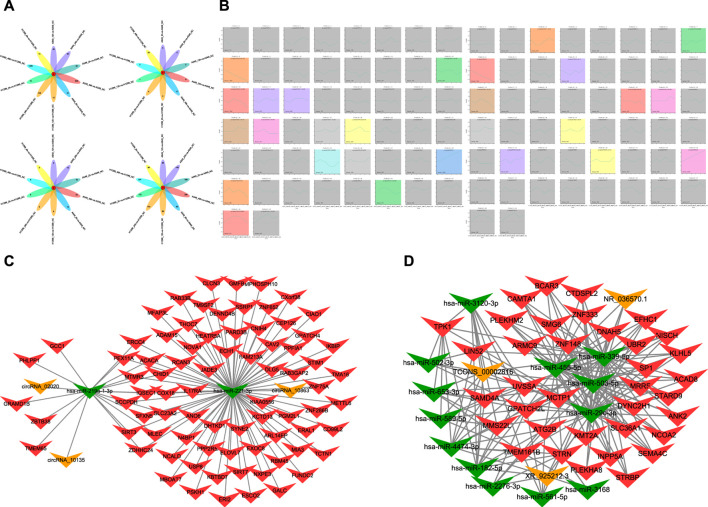
**(A)** Diagram of intersections in different mRNA, miRNA, lncRNA, and circRNA after radiation of A549 and H1299 cells in five time points (2, 6, 12, 24, and 48 h) compared with non-irradiated cells. **(B)** Trend chart of mRNA after radiotherapy in A549 and H1299 cells in STEM analyses (a screenshot of the main interface window of the STEM is found in Figure 1B. In this window, each box corresponds to one of the model temporal expression profiles. The data were sampled at five time points 2, 6, 12, 24, and 48 h. The number at the top of a profile box is the profile ID number. The colored profiles had a statistically significant number of genes assigned). **(C)** Significant ceRNA network related to five time points (2, 6, 12, 24, and 48 h) after radiation therapy in A549 and H1299 cells: mRNA–miRNA–circRNA network. **(D)** Significant ceRNA network related to five time points (2, 6, 12, 24, 48 h) after radiation therapy in A549 and H1299 cells: mRNA–miRNA–lncRNA network.

The analysis samples were analyzed with the Short Time-series Expression Miner (STEM) (Ernst and Bar-Joseph, 2006) in the order of [“A549_NC”—> “A549_2 h”—> “A549_6 h”—> “A549_12 h”—> “A549_24 h”—> “A549_48 h”]. The *p*-value was corrected by the false discovery rate method, and the significant modules with *p*-value less than 0.05 were selected. A total of 16 significant modules of A549 were screened in 50 modules. A total of 39 significant modules were also screened in the order of ["H1299_NC”—> “H1299_2 h”—> “H1299_6 h”—> “H1299_12 h”—> “H1299_24 h”—> “H1299_48 h”]. The trend map and clustering heatmap of the significant module in A549 and H1299 were drawn (Figure 1B). These mRNAs in significant modules related to time after radiation were recorded as STEM genes.

The intersection between the NCdiffmRNA and STEM gene was taken, named as diffmRNA. DiffmRNA represented differences after radiation in 48 h caused by the two cell lines. The remaining common gene was named commonmRNA. CommonmRNA represents common genes of non-small cell lung cancer, regardless of the differences caused by the two cell lines after radiation in 48 h. By applying the same process for NCdiffcircRNA, NCdifflncRNA, and NCdiffmiRNA, we got commoncircRNA, commonlncRNA, and commonmiRNA, respectively.

#### 2.6.3 Ingenuity pathway analysis

CommonmRNA at 2, 6, 12, 24, and 48 h was analyzed by Ingenuity pathway analysis (IPA, http://www.ingenuity.com). The setting is shown in Supplementary Materials (S2–S6).

#### 2.6.4 CeRNA analysis and enrichment analysis

Pearson r was used to calculate the correlation of 24 samples. MiRNA–mRNA relationship pairs were screened (the absolute correlation coefficient value greater than or equal to 0.60, and the *p*-value was less than or equal to 0.05). According to the mechanism of action of miRNA and mRNA, the relationship pairs of negative regulation were screened, and miRNA–mRNA relationship pairs were screened. The miRanda program was used to predict the binding between these miRNA–mRNA sequences, using the default parameter of miRanda v3.3a. Finally, pairs of miRNA–mRNA relationships were obtained. Pairs of miRNA–circRNA relationships were also obtained by the same way.

For these predicted relationships, the MuTaME method was performed to get a ceRNA score (Tay et al., 2011). At the same time, the *p*-value corresponding to the ceRNA relationship was calculated in combination with the hypergeometric distribution, and the smaller the *p*-value, the more significant these miRNAs shared between the two ceRNAs (mRNA and target).

MRNA–circRNA relationship pairs was screened by Pearson r (the absolute correlation coefficient value greater than or equal to 0.60, and the *p*-value was less than or equal to 0.05). According to the role of mRNA–circRNA in the ceRNA relationship, the relationship between mRNA and circRNA with positive correlation was screened, and the results of the ceRNA score were calculated and the two intersected. Then, the ceRNA results helped build the ceRNA network.

GO and KEGG pathway analyses were performed on the mRNA in the ceRNA network. The top 30 mRNAs in the RNA score in the mRNA–miRNA–circRNA network and the mRNA–miRNA–lncRNA network are used for pathway enrichment by GO and KEGG analyses, separately. CeRNA analysis and enrichment analysis of mRNA gene sets helped obtain key regulatory network molecules and key pathways that may be caused by radiation in 48 h.

### 2.7 Statistical analysis

Line charts and histograms were produced by GraphPad 7.0. Bioinformatics analysis was carried out using the R language (Version 4.0.0). The gray value of protein bands was analyzed by ImageJ software, and statistical analysis was carried out using SPSS 24.0. Also, the comparison of two sets of disordered variables was *t*-tested; the categorical variables were chi-squared. The bilateral *p* < 0.05 was statistically significant.

## 3 Results

### 3.1 Whole-transcriptome sequencing of NSCLC cells

A flowchart is shown in Supplementary Materials S1. The differential mRNA of the no radiation group of A549 and H1299 (NCdiffmRNA) has 5452 genes, which is related to the intrinsic difference between the two cell lines. A total of 2755 genes were upregulated and 2697 downregulated in NCdiffmRNA. The STEM intersection gene is composed of 5076 genes, which is associated with time change after radiation within 48 h. Intersecting diffmRNA has 576 genes, related to the intrinsic difference of two cell lines and time change after radiation. CommonmRNA has 4509 genes, which is related to the time change after radiation within 48 h in non-small cell lung cancer.

The whole-transcriptome sequencing results of A549 and H1299 showed that the intersections of different mRNA at different times are 0 compared with non-irradiated cells. The differential mRNAs at 2, 6, 12, 24, and 48 h of A549, compared with the no radiation group, are 40 genes, 27 genes, 26 genes, 84 genes, and 509 genes, respectively. The differential mRNAs at 2, 6, 12, 24, and 48 h of H1299, compared with the no radiation group, are 15 genes, 14 genes, 15 genes, 109 genes, and 1295 genes, respectively. The results of the intersection of differential miRNAs, lncRNAs, and circRNAs are 0, suggesting that the genome is in a significant dynamic change within 48 h after radiation in NSCLC (Figure 1A).

The differential lncRNAs at 2, 6, 12, 24, and 48 h in A549 are 168 lncRNAs, 80 lncRNAs, 96 lncRNAs, 140 lncRNAs, and 256 lncRNAs, respectively. The differential lncRNAs at 2, 6, 12, 24, and 48 h in H1299 are 123 lncRNAs, 86 lncRNAs, 86 lncRNAs, 143 lncRNAs, and 331 lncRNAs, respectively. The results of the difference between miRNAs and circRNAs are shown in Figure 1A.

### 3.2 The STEM analysis of temporal trends of mRNAs

A total of 16 significant modules were screened in A549, and 13 significant modules were screened in H1299. We plotted a meaningful module trend map (Figure 1B) and took the union of genes in 39 modules. Finally, we got 5076 genes in 39 modules related to the temporal changes in gene expression after radiation in NSCLC.

### 3.3 CeRNA analyses and enrichment analyses

**FIGURE 2:**
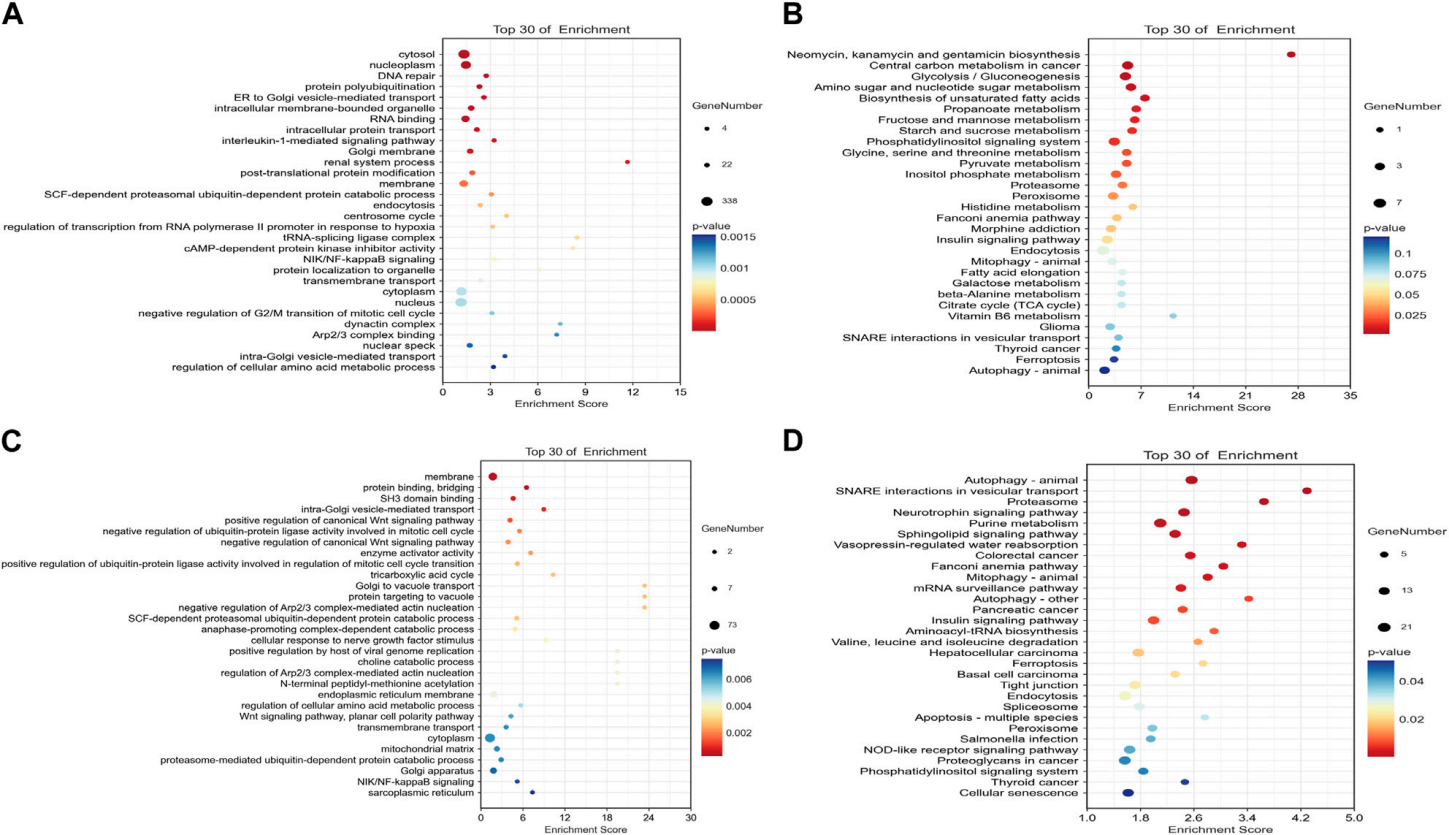
Dot plots of the top 30 mRNAs in the ceRNA network. **(A)** Significantly different pathways from GO analysis in the mRNA–miRNA–circRNA network. **(B)** Significantly different pathways from KEGG analyses in the mRNA–miRNA–circRNA network. **(C)** Significantly different pathways from GO analysis in the mRNA–miRNA–lncRNA network. **(D)** Significantly different pathways from KEGG analysis in the mRNA–miRNA–lncRNA network.

The ceRNA score is used to obtain the mRNA–miRNA–lncRNA network (Figure 1C), and hsa-miR-503-5p, hsa-miR-455-5p, hsa-miR-29c-3p, and hsa-miR-339-5p are located at the core of ceRNA. GO analysis showed that evident biological processes include DNA repair, negative regulation of G2/M transition of the mitotic cell cycle, protein polyubiquitination, ER to Golgi vesicle-mediated transport, and intracellular protein transport. KEGG analyses showed that evident pathways include autophagy, ferroptosis, endocytosis, purine metabolism, neurotrophin signaling pathway, and insulin signaling pathway (Figures 2A, B). Similarly, in the mRNA–miRNA–circRNA network (Figure 1D), miR-219-1-3p and miR-221-3p are in the core. GO analysis showed that evident biological processes include the intra-Golgi vesicle-mediated transport, positive regulation of the canonical Wnt signaling pathway, negative regulation of Arp2/3 complex-mediated actin nucleation, the SCF-dependent proteasomal ubiquitin-dependent protein catabolic process, and regulation of the Arp2/3 complex-mediated actin nucleation. KEGG analyses showed evident pathways including SNARE interactions in vesicular transport, ferroptosis, autophagy in animals, and apoptosis (Figures 2C, D).

### 3.4 Ingenuity pathway analysis

**FIGURE 3:**
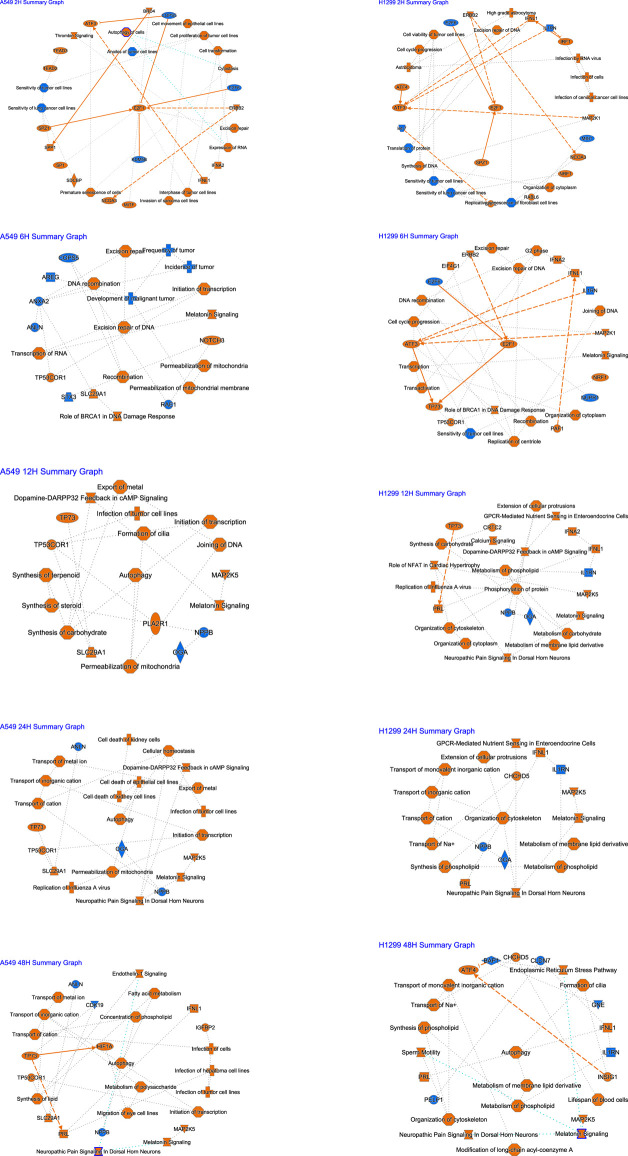
Summary of IPA functions for radiotherapy of A549 and H1299 cells at five time points (2, 6, 12, 24, and 48 h).

A graphical summary (Figure 3) showed that E2F1 regulation occupies a key position at 2 h after radiation, damage repair of DNA at 6 h accounts for the core, and autophagy occupies the core at 12–48 h in A549. In H1299, E2F1 regulation within 2–6 h after radiation occupies the core position, and cellular changes within 12–24 h are mainly related to metabolism; autophagy occupies the core position at 48 h.

**FIGURE 4:**
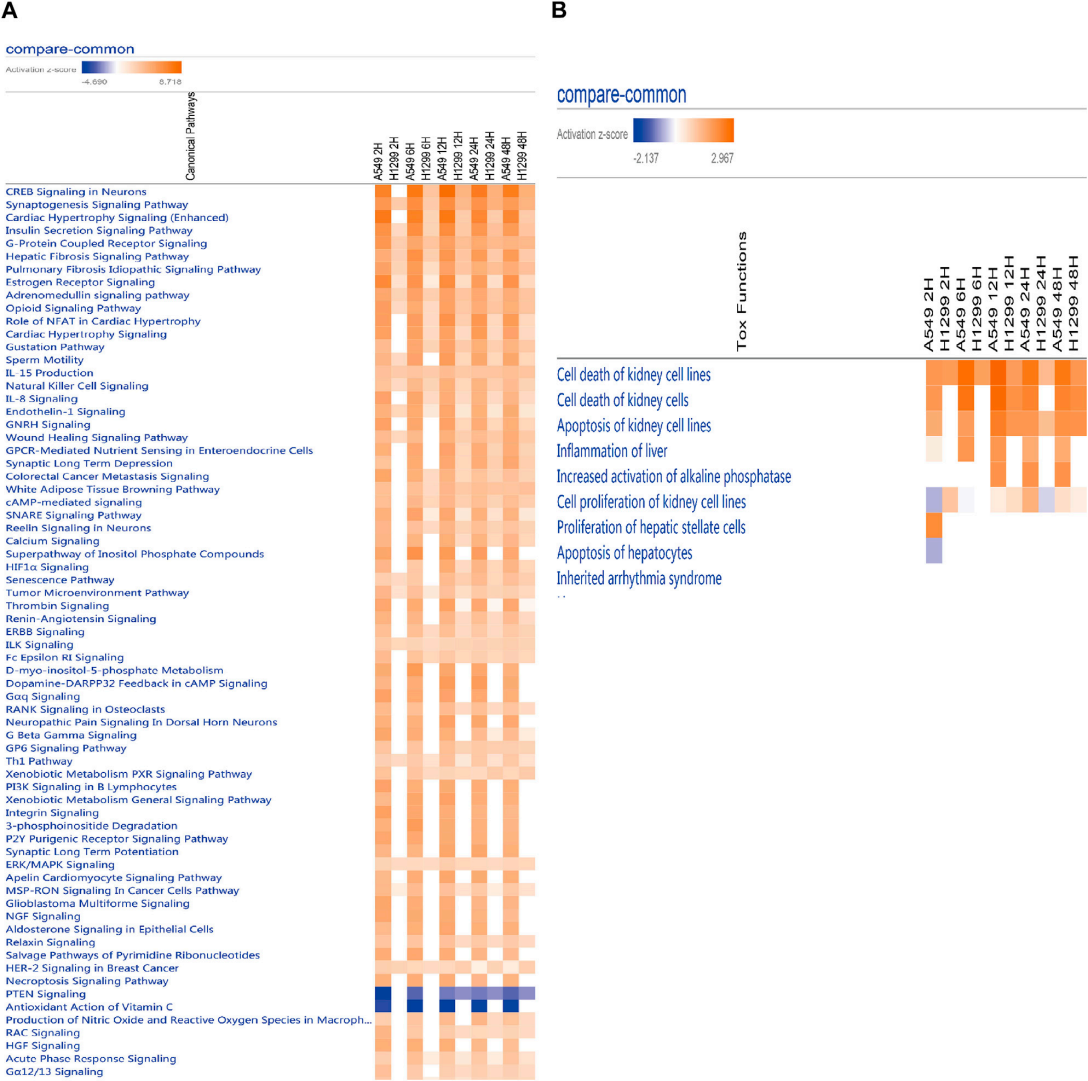
**(A)** Heatmap of the classical pathway trend of A549 and H1299 cells at five time points (2, 6, 12, 24, and 48 h) in radiotherapy predicted by IPA. **(B)** Heatmap of the toxicity pathway trend of A549 and H1299 cells at five time points (2, 6, 12, 24, and 48 h) in radiotherapy predicted by IPA.

The analysis of causal pathways (Figure 4A) shows evident pathways and significant changes caused by radiation including CREB signaling in neurons and synaptogenesis signaling pathway, cardiac hypertrophy signaling (enhanced), insulin secretion signaling pathway, G-protein coupled receptor signaling, hepatic fibrosis signaling pathway, and pulmonary fibrosis idiopathic signaling pathway.

Multi-time analysis of toxic pathways (Tox functions) is shown in Figure 4B. The results show that evident pathways are cell death of kidney cell lines, cell death of kidney cells, apoptosis of kidney cell lines, inflammation of the liver, increased activation of alkaline phosphatase, cell proliferation of kidney cell lines, proliferation of hepatic stellate cells, and apoptosis of hepatocytes, which suggests liver and kidney death, or the damage caused by radiation with 10 Gy is more evident.

Diseases/biological functions involving different genes/proteins over multiple periods are shown in Table 1. The top 10 pathways are infection of cells, transport of molecules, viral infection, migration of cells and cell movement, infection of tumor cell lines, metabolism of carbohydrate, synthesis of carbohydrate, infection by the RNA virus, and protein kinase cascade.

**TABLE 1:** Top 30 diseases and functions with the most significant changes at five time points (2, 6, 12, 24, and 48 h) after radiation in NSCLC.

Diseases and biofunctions	A549 2 h	H1299 2 h	A549 6 h	H1299 6 h	A549 12 h	H1299 12 h	A549 24 h	H1299 24 h	A549 48 h	H1299 48 h
Infection of cells	10.645	N/A	11.243	N/A	N/A	N/A	9.581	N/A	8.77	N/A
Transport of molecules	5.234	2.422	N/A	3.109	5.675	3.297	5.069	3.144	5.302	3.683
Viral infection	11.546	N/A	N/A	N/A	N/A	N/A	9.767	N/A	9.214	N/A
Migration of cells	N/A	4.655	N/A	3.543	N/A	5.121	N/A	5.455	N/A	5.818
Cell movement	N/A	4.822	N/A	N/A	N/A	5.644	N/A	5.904	N/A	6.204
Infection of tumor cell lines	N/A	N/A	N/A	N/A	7.873	N/A	7.721	N/A	6.915	N/A
Metabolism of carbohydrate	4.247	N/A	4.093	N/A	4.419	1.067	4.081	N/A	4.316	N/A
Synthesis of carbohydrate	4.292	N/A	4.141	N/A	4.464	N/A	4.129	N/A	4.362	N/A
Infection by the RNA virus	10.769	N/A	N/A	N/A	N/A	N/A	10.216	N/A	N/A	N/A
Protein kinase cascade	4.53	2	N/A	N/A	4.199	N/A	4.222	N/A	4.721	N/A
Fatty acid metabolism	N/A	2.923	N/A	3.088	N/A	3.508	N/A	3.508	3.36	3.232
Synthesis of lipids	N/A	1.387	N/A	2.402	4.985	2.013	4.047	N/A	4.555	N/A
Invasion of cells	N/A	3.745	N/A	N/A	N/A	5.101	N/A	4.972	N/A	5.376
Cell movement of tumor cell lines	N/A	4.09	N/A	N/A	N/A	4.446	N/A	4.549	N/A	4.982
Migration of tumor cell lines	N/A	4.356	N/A	N/A	N/A	4.159	N/A	4.266	N/A	4.859
Extracranial solid tumors	2.991	−1.342	2.104	−1.067	1.918	−1.633	2.217	−1.134	1.453	−1.195
Migration of endothelial cells	N/A	2.722	N/A	2.926	N/A	3.223	N/A	3.505	N/A	3.74
Oxidation of lipids	N/A	N/A	N/A	N/A	3.404	2.407	3.112	2.407	3.101	N/A
Invasion of tumor cell lines	N/A	3.732	N/A	N/A	N/A	5.013	N/A	N/A	N/A	5.315
Cell proliferation of tumor cell lines	6.878	N/A	N/A	N/A	N/A	2.362	N/A	2.064	N/A	2.314
Malignant solid tumors	1.787	−1.195	1.28	−1.698	1.295	−1.195	1.143	−1.195	1.058	−1.51
Autophagy	3.156	N/A	1.378	N/A	2.22	N/A	2.942	N/A	3.067	N/A
Metabolism of polyunsaturated fatty acids	N/A	2.582	N/A	2.433	N/A	2.582	N/A	2.582	N/A	2.582
Organization of the cytoplasm	N/A	N/A	N/A	N/A	3.967	N/A	3.911	N/A	3.949	N/A
Cellular homeostasis	3.896	N/A	N/A	N/A	N/A	N/A	3.96	N/A	3.62	N/A
Solid tumors	2.954	N/A	1.883	N/A	1.967	N/A	2.191	N/A	2.151	N/A
Cell death of tumor cell lines	−4.481	−1.916	N/A	N/A	N/A	−1.469	N/A	−1.442	N/A	−1.77
Metabolism of membrane lipid derivatives	2.564	N/A	N/A	N/A	2.947	N/A	2.641	N/A	2.918	N/A
Replication of Influenza A virus	N/A	N/A	N/A	N/A	N/A	N/A	5.534	N/A	5.439	N/A
Synthesis of polysaccharides	2.596	N/A	N/A	N/A	2.578	N/A	2.377	1	2.399	N/A

At 2 h after radiotherapy, the top canonical pathways in NSCLC mainly include the BAG2 signaling pathway, the FAT10 signaling pathway, and inhibition of ARE-mediated mRNAs. Top diseases and biofunctions mainly include cancer, organismal injury and abnormalities, endocrine system disorders, and gastrointestinal diseases. Molecular and cellular functions focus on DNA replication, recombination and repair, and cell death and survival (Supplementary Materials S2). At 6 h after radiotherapy, top canonical pathways in NSCLC mainly include the BAG2 signaling pathway and the FAT10 signaling pathway. The results of diseases and biofunctions and molecular and cellular functions are similar with those of NSCLC at 2 h (Supplementary Materials S3).

At 12 h after radiotherapy, top canonical pathways in NSCLC mainly include the CLEAR signaling pathway and melatonin signaling. Top canonical pathways in NSCLC include neurological diseases, compared with the results at 2 h and 6 h. Molecular and cellular functions focus on cellular assembly and organization, cell cycle, and carbohydrate metabolism (Supplementary Materials S4).

At 24 h after radiotherapy, top canonical pathways in NSCLC mainly include the super pathway of cholesterol biosynthesis, cholesterol biosynthesis I, and cholesterol biosynthesis II (*via* 24,25-dihydrolanosterol). Top canonical pathways are the same with the pathways at 12 h. Molecular and cellular functions mainly focus on the metabolism (Supplementary Materials S5).

At 48 h, top canonical pathways in NSCLC mainly include the CLEAR signaling pathway. Top canonical pathways are the same with the pathways at 12 and 24 h. Molecular and cellular functions mainly focus on the metabolism (Supplementary Materials S6).

### 3.5 Cell cycle analysis

**FIGURE 5:**
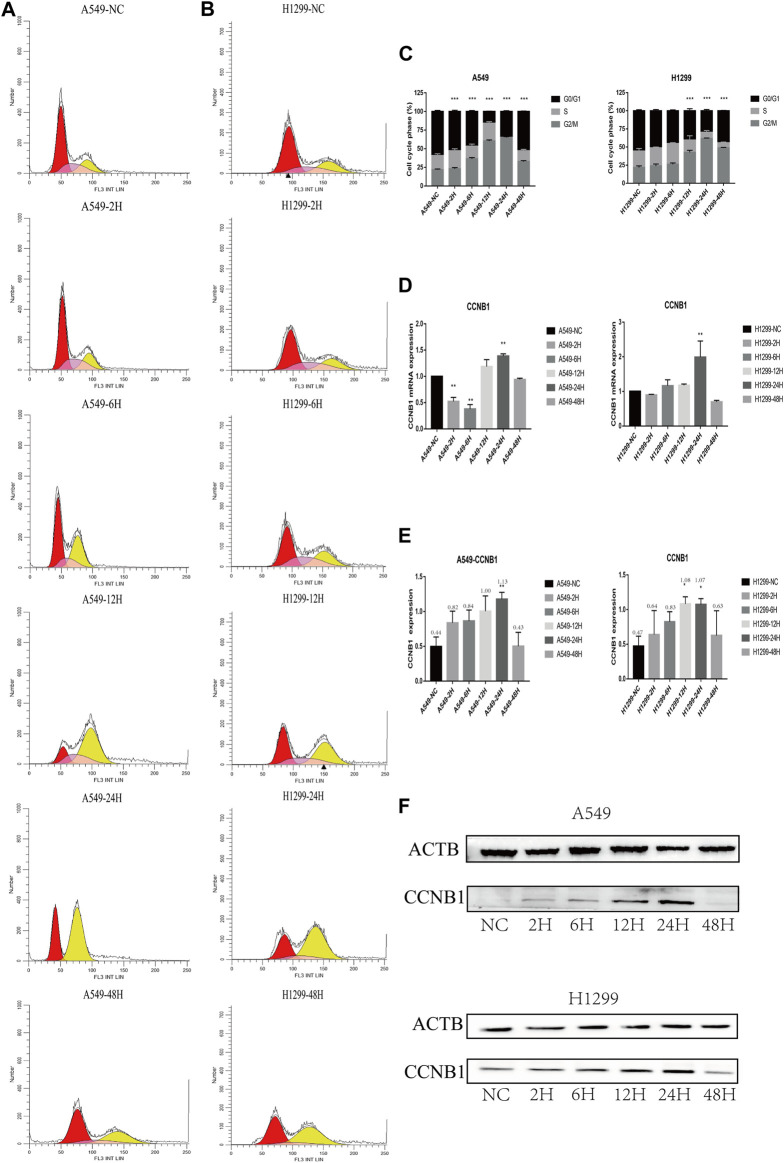
Cell cycle and cell cycle-related protein expression after radiation in NSCLC cells. *p < 0.05, **p < 0.01, and ***p < 0.001. **(A)** Cell cycle analysis of A549 at 2, 6, 12, 24, and 48 h after radiation. **(B)** Cell cycle analysis of H1299 at 2, 6, 12, 24, and 48 h after radiation. **(C)** Statistical analysis of different phases of the cell cycle at 2, 6, 12, 24, and 48 h after radiation in A549 and H1299 cell lines. **(D)** PCR statistical results of CCNB1 at 2, 6, 12, 24, and 48 h after radiation in A549 and H1299 cell lines. **(E)** Western blot statistical results of cyclin B1 at 2, 6, 12, 24, and 48 h after radiation in A549 and H1299 cell lines (quantitative data are shown as means). **(F)** Western blot results of cyclin B1 at 2, 6, 12, 24, and 48 h after radiation in A549 and H1299 cell lines, compared with the non-radiation control group.

The proportion of each cycle phase is shown in A, B, and C in Figure 5. Compared with the non-radiation group, the G2/M stage arrest of post-radiation NSCLC gradually worsened, peaking at 24 h, and decreased progressively at 48 h. Changes in cyclin B1 (CCNB1) showed a similar trend. The PCR results show a maximum value was reached at 24 h, see Figure 5D, and the WB results also show the expression of CCNB1 reached a maximum at 24 h (Figures 5E, F).

## 4 Discussion

Precision medicine is becoming a new direction for cancer treatment.

Personalized and precise management relies heavily on developing new technologies for next-generation sequencing and data processing of radiobiological information (Yang et al., 2020).

In this study, whole-transcriptome sequencing was used to comprehensively detect molecular changes of NSCLC in different periods after radiation, providing a dynamic molecular process map for precision radiotherapy.

Data from short time-series expressions can be analyzed using two methods. The first employs methods that do not take advantage of the sequential information in time-series data. The second method was primarily designed for a longer time series, ignoring the temporal dependency among successive time points. The Short Time-series Expression Miner was designed for short time-series microarray gene expression data. It also has the advantage of visualization capabilities and integration with GO (Ernst and Bar-Joseph, 2006).

Time trend analysis obtained significant dynamic changes in mRNA, miRNA, lncRNA, and circRNA gene sets. According to the ceRNA analysis of RNAs related to time, we found the main regulatory networks and key molecules of post-radiation in NSCLC. These can provide new ideas for post-radiation molecular regulation mechanism research and seeking to target molecular therapies for NSCLC. For example, in the ceRNA network, miR-219-1-3p, which occupies the core, negatively regulates MUC4 and has a tumor-suppressive effect in pancreatic cancer (Chae et al., 2017). Related studies have found that miR-219-1-3p inhibits proliferation and weakens cell migration (Lahdaoui et al., 2015). MiR-221-3p downregulates the proto-oncogene MDM2, reversing paclitaxel resistance in non-small cell carcinoma and inducing apoptosis (Ni et al., 2021).

The results of GO and KEGG include DNA repair, negative regulation of G2/M transition of mitotic cell cycle, regulation of the autophagosome assembly, DNA replication, autophagy, ferroptosis, apoptosis, glucose metabolism, and insulin pathways. It broadens the content of radiobiology and the study of intersecting fields, providing new insights for combining radiation and drugs to improve radiotherapy efficacy.

IPA implies NSCLC cells started DNA damage and repair mainly in the early phase (2–6 h) after radiation, and E2F1 may play an important role in this early response phase. The cells started autophagy mainly in the later stages (24–48 h). These findings significantly enrich the content of radiobiology at various periods and help us get the key molecular or pathway or function to respond to radiotherapy at a specific time slot. Additionally, the molecules we are familiar with may regulate other pathways under radiotherapy conditions, which open up our perspective of molecular biology. For example, it is acknowledged that E2F1 is related to the cell cycle (Schuldt, 2011). In recent years, RB/E2F1 has been the main regulator of cancer cell metabolism in advanced diseases. It promotes the synthesis of antioxidant glutathione after RB loss, regulates redox metabolism, and reveals the protective effect of therapeutic intervention on reactive oxygen species (Mandigo et al., 2021). E2F1 may also be associated with the metabolism after radiotherapy by combining IPA, GO, and KEGG results, but it needs to be verified experimentally.

Toxic pathways after radiation mainly focus on hepatic and renal pathways. The in-depth understanding of the molecular and pathophysiology of radiation organs needs further study (Wang and Tepper, 2021).

The G2-phase arrest plays a role in cell survival after irradiation (Hwang and Muschel, 1998). Cells at this stage are sensitive to radiation therapy. Some studies discuss the potential use of G2/M cell cycle checkpoint inhibitors to enhance tumor control rates (Hellmann and Rhomberg, 1991; Löbrich and Jeggo, 2007; Dillon et al., 2014). Our results suggest that 24 h is proper for radiation therapy to maximize the effect of killing tumor cells. Some studies showed that A549 under dose 2 Gy at G2 / M phase arrest the most at 72 h (Yang et al., 2015). Our results suggest that 24 h may be best for radiation therapy in larger doses (10 Gy), guiding the practice of clinical radiation, combination chemotherapeutic drugs, and radiotherapy sensitizers.

There are few papers that compare the changes in transcriptome induced by low-dose radiation with those induced by high-dose SBRT radiation. Research about chronic low-dose radiation exposure in a zebrafish model found that radiation exposure resulted in transcriptomic perturbations in wound healing, immune response, lipid metabolism and absorption, and fibrogenic pathways (Cahill et al., 2023). Genomic and transcriptomic results of SBRT showed that in patients with renal cell carcinoma, pathways including G2/M checkpoint, mitotic spindle, and E2F targets were significant (Zengin et al., 2023). These results are consistent with our results.

Tumor treating fields (TTFields) is a new modality of cancer treatment. The treatment is based on transdermally transmitting alternating current (AC) electric fields at 100–400 kHz to tumors with two orthogonal transducer arrays (Moser et al., 2022). It can cause DNA damage and replication stress (Karanam et al., 2020). Our results can be combined with those of tumor treating fields to provide a biological basis for the timing of tumor treating fields after SBRT for non-small cell lung cancer. Compared with a low dose, our results would provide more economical ways to apply to the TTF. At the same time, our research further screens and models the common non-small cell lung cancer genes, which can achieve individualized treatment for patients with high matching genes with our gene set.

There were some flaws in the experiment. Our selection of genes common to non-small cell lung cancer needs to be verified. The genes and networks that change in each period need to be further explored. We did not perform animal experiments and lacked clinical samples of radiation therapy to verify whether the results we found were related to radiation. Further research is needed in the future.

## 5 Conclusion

Our transcriptomic and experimental analyses provide the dynamic change of radiation therapy in NSCLC, enriching the content of radiobiology in precision radiation oncology.

## Data Availability

The original contributions presented in the study are publicly available. This data can be found here: https://www.ncbi.nlm.nih.gov/ [Accession number PRJNA916274]. Due to disk damage, data of files (A549_2h2.R1.fastq.gz A549_2h2.R2.fastq.gz. H1299_NC2.R1.fastq.gz H1299_NC2.R2.fastq.gz A549_48h2.R1.fastq.gz, A549_NC1.R1.fastq.gz A549_NC1.R2.fastq.gz H1299_2h2.R1.fastq.gz) are unavailable. Further enquiries can be directed to the corresponding author.

## Supplementary material

SUPPLEMENT MATERIALS S1 Flowchart of genome-wide analysis of human non-small cell lung cancer after radiation

SUPPLEMENT MATERIALS S2 IPA results of A549 and H1299 after radiation at 2 h

SUPPLEMENT MATERIALS S3 IPA results of A549 and H1299 after radiation at 6 h

SUPPLEMENT MATERIALS S4 IPA results of A549 and H1299 after radiation at 12 h

SUPPLEMENT MATERIALS S5 IPA results of A549 and H1299 after radiation at 24 h

SUPPLEMENT MATERIALS S6 IPA results of A549 and H1299 after radiation at 48 h
