# Supplementary figures and images for: Genome-wide analyses of lung cancer after single high-dose radiation at five time points (2, 6, 12, 24, and 48 h)
Source: Front Genet. 2023 Mar 3;14:1126236. doi: 10.3389/fgene.2023.1126236 (PMC10020487; doi:10.3389/fgene.2023.1126236)

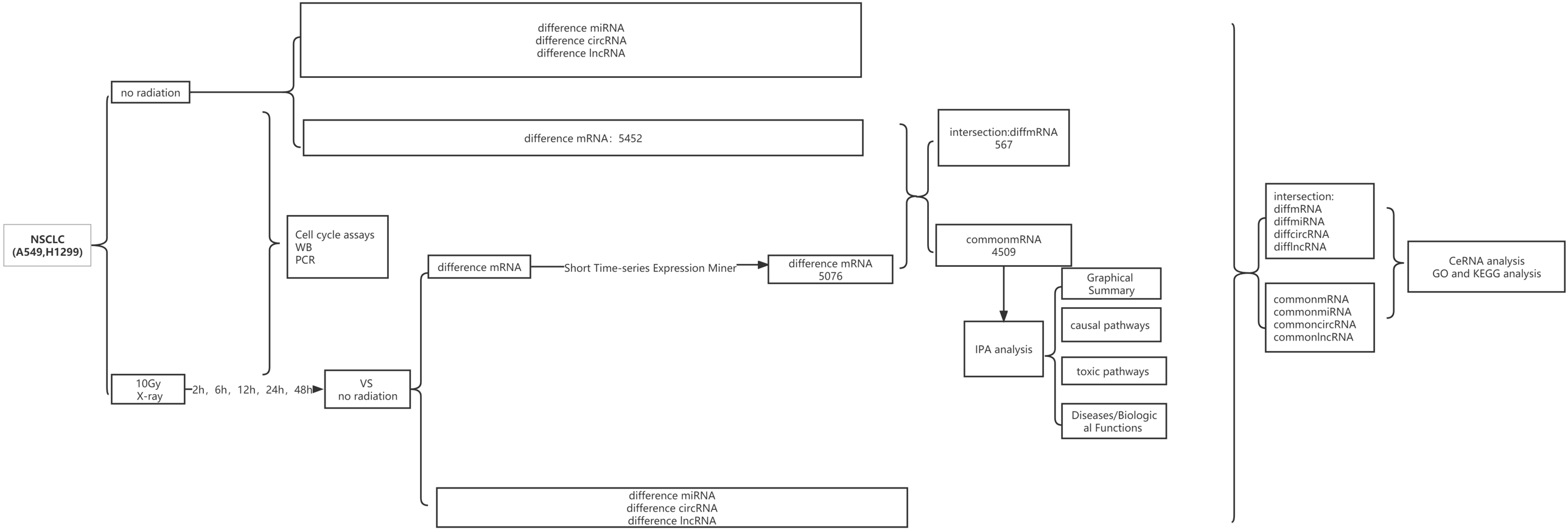

Supplement: Supplementary file 6 [file DataSheet1.pdf]
