# Supplementary material for: Genome-wide analyses of lung cancer after single high-dose radiation at five time points (2, 6, 12, 24, and 48 h)
Source: Front Genet. 2023 Mar 3;14:1126236. doi: 10.3389/fgene.2023.1126236 (PMC10020487; doi:10.3389/fgene.2023.1126236)
Supplement: Supplementary file 7 [file DataSheet5.pdf]

INGENUITY
PATHWAY ANALYSIS

Analysis Name: A549 24H
Analysis Creation Date: 2022-01-13
Build version: exported
Content version: 70750971 (Release Date: 2021-10-22)

Experiment Metadata

Name	Value

Analysis Settings

Reference set: Ingenuity Knowledge Base (Genes + Endogenous Chemicals)
Relationship to include: Direct and Indirect
Includes Endogenous Chemicals
Optional Analyses:

Filter Summary:
Consider only molecules and/or relationships where
(species = Human) AND
(tissues/cell lines = Fibroblasts OR RKO OR Other Ovarian Cancer Cell Lines OR Other Cells OR Other Colon Cancer Cell Lines OR OVCAR-3 OR Striatum OR Min6 OR 3T3-L1 cells OR HOP-62 OR Jurkat OR OVCAR-5 OR Prostate Gland OR WEHI-231 OR Central memory cytotoxic T cells OR Other Smooth muscle cells OR Stromal cells OR Chondrocytes OR Neutrophils OR HS 578T OR CD56dim NK cells OR SF-295 OR Lymphoma Cell Lines not otherwise specified OR Effector memory helper T cells OR Pancreas OR Activated helper T cells OR Memory T

(c) 2000-2022 QIAGEN. All rights reserved.

INGENUITY PATHWAY ANALYSIS

lymphocytes not otherwise specified OR RAW 264.7 OR Corpus Callosum OR Central memory helper T cells OR Pheochromocytoma cell lines not otherwise specified OR Esophagus OR Other Organ Systems OR Keratinocytes OR Large Intestine OR Other CNS Cell Lines OR Peritoneal macrophages OR Effector memory cytotoxic T cells OR Salivary Gland OR Monocytes not otherwise specified OR HepG2 OR HT29 OR Other Myeloma Cell Lines OR Thalamus OR Stomach OR J774 OR Lymph node OR Mesenchymal stem cells OR Skeletal Muscle OR MDA-MB-361 OR Activated CD56dim NK cells OR Placenta OR Ovary OR MALME-3M OR Neurons not otherwise specified OR Other Bone marrow cells OR Peripheral blood lymphocytes OR UACC-257 OR Other Tissues and Primary Cells OR Other Neurons OR SF-268 OR Smooth muscle cells not otherwise specified OR White Matter OR NCI-H332M OR Other Monocyte-derived dendritic cells OR Cos-7 cells OR U2OS OR Myeloma Cell Lines not otherwise specified OR Dendritic cells not otherwise specified OR HCT-116 OR Intraepithelial T lymphocytes OR OVCAR-8 OR OVCAR-4 OR Amygdala OR Vd2 Gamma-delta T cells OR U251 OR Hematopoietic progenitor cells OR Cells not otherwise specified OR Other Breast Cancer Cell Lines OR NCI-H226 OR Other Dendritic cells OR Plasma cells OR THP-1 OR Forestomach OR Mononuclear leukocytes not otherwise specified OR HuH7 OR Other Memory T lymphocytes OR Purkinje cells OR Activated CD56bright NK cells OR Granule cells OR Cerebral Cortex OR Other Stem cells OR Nervous System not otherwise specified OR BT-549 OR Embryonic stem cells OR A375 OR Stem cells not otherwise specified OR A549-ATCC OR Caudate Nucleus OR Cerebral Ventricles OR HL-60 OR Eosinophils OR HUVEC cells OR Other Mononuclear leukocytes OR HeLa OR NB4 OR Splenocytes OR INS-1 OR Other Epithelial cells OR Cervical cancer cell line not otherwise specified OR Other Macrophage Cancer Cell Lines OR Other B lymphocytes OR Sertoli cells OR Epidermis OR Lung OR Prostate Cancer Cell Lines not otherwise specified OR SN12C OR Kidney cell lines not otherwise specified OR Osteosarcoma Cell Lines not otherwise specified OR T47-D OR H460 OR Other Cell Line OR PC-3 OR Pre-B lymphocytes OR Hypothalamus OR Pituitary Gland OR Vascular smooth muscle cells OR SR OR RBL-2H3 OR Bone marrow-derived macrophages OR Thymocytes OR Other Immune cells OR PBMCs OR Other Lung Cancer Cell Lines OR Thyroid Gland OR J-774A.1 OR BDCA-1+ dendritic cells OR Trigeminal Ganglion OR Other Lymphoma Cell Lines OR SF-539 OR Ovarian Cancer Cell Lines not otherwise specified OR Pyramidal neurons OR SW-480 OR HMC-1 OR PANC-1 OR Substantia Nigra OR U87MG OR CD34+ cells OR Cornea OR Dermis OR LNCaP cells OR SK-MEL-28 OR MDA-N OR Putamen OR Effector T cells OR Kidney Cancer Cell Lines not otherwise specified OR KM-12 OR Granulocytes not otherwise specified OR Murine NKT cells OR UO-31 OR Other Macrophages OR Subventricular Zone OR Spleen OR Mature monocyte-derived dendritic cells OR Myeloid dendritic cells OR Other Melanoma Cell Lines OR Immune cells not otherwise specified OR IGROV1 OR Other Cervical cancer cell line OR Retina OR Osteoblasts OR Nucleus Accumbens OR Other Fibroblast cell lines OR Smooth Muscle OR Plasmacytoid dendritic cells OR K-562 OR Other Leukemia Cell Lines OR Other Teratocarcinoma Cell Lines OR SW-620 OR Endothelial cells not otherwise specified OR Bladder OR Kidney OR Adrenal Gland OR HEL OR Pro-B lymphocytes OR Cortical neurons OR Gray Matter OR Macrophages not otherwise specified OR NCI-ADR-RES OR Teratocarcinoma Cell Lines not otherwise specified OR Dorsal Root Ganglion OR Mast cells OR Naive B cells OR Thymus OR Neuroblastoma Cell Lines not otherwise specified OR Other Osteosarcoma Cell Lines OR Pancreatic Cancer Cell Lines not otherwise specified OR Other Monocytes OR SK-MEL-5 OR

(c) 2000-2022 QIAGEN. All rights reserved.

A498 OR Fibroblast cell lines not otherwise specified OR Microglia OR Other Kidney cell lines OR Epithelial cells not otherwise specified OR 786-0 OR Blood platelets OR Small Intestine OR Peripheral blood leukocytes not otherwise specified OR Cerebellum OR Astrocytes OR ACHN OR Testis OR Cell Line not otherwise specified OR U266 OR NCI-H522 OR Trachea OR Swiss 3T3 cells OR Th1 cells OR MDA-MB-435 OR Other Hepatoma Cell Lines OR NIH/3T3 cells OR Other Kidney Cancer Cell Lines OR Th17 cells OR MDA-MB-231 OR Natural T-regulatory cells OR Other Peripheral blood leukocytes OR Other Neuroblastoma Cell Lines OR PC-12 cells OR Other T lymphocytes OR Adipocytes OR Adipose OR HOP-92 OR Hepatoma Cell Lines not otherwise specified OR HCC-2998 OR Bone marrow-derived dendritic cells OR Sciatic Nerve OR Memory B cells OR NK cells not otherwise specified OR Other Pheochromocytoma cell lines OR Calvaria OR CCRF-CEM OR Hippocampus OR Colon Cancer Cell Lines not otherwise specified OR Mammary Gland OR Spinal Cord OR CAKI-1 OR NT2/D1 OR Monocyte-derived macrophage OR BA/F3 OR MCF7 OR Immature monocyte-derived dendritic cells OR Hepatocytes OR Effector memory RA+ cytotoxic T cells OR UACC-62 OR Organ Systems not otherwise specified OR Other Granulocytes OR Ventricular Zone OR Leukemia Cell Lines not otherwise specified OR CNS Cell Lines not otherwise specified OR Liver OR Vd1 Gamma-delta T cells OR Beta islet cells OR Lens OR LOX IMVI OR M14 OR Melanocytes OR Bone marrow cells not otherwise specified OR DU-145 OR U937 OR Monocyte-derived dendritic cells not otherwise specified OR Langerhans cells OR Megakaryocytes OR Uterus OR Granulosa cells OR COLO205 OR Immune cell lines not otherwise specified OR CD56bright NK cells OR Lymphocytes not otherwise specified OR Melanoma Cell Lines not otherwise specified OR Brainstem OR Microvascular endothelial cells OR Choroid Plexus OR Granule Cell Layer OR Other Immune cell lines OR Hep3B OR BDCA-3+ dendritic cells OR T lymphocytes not otherwise specified OR Olfactory Bulb OR Tissues and Primary Cells not otherwise specified OR SK-OV-3 OR Other Lymphocytes OR SK-MEL-2 OR Other Endothelial cells OR Oocytes OR Naive helper T cells OR MG-63 OR Cartilage Tissue OR Macrophage Cancer Cell Lines not otherwise specified OR P19 OR MDA-MB-468 OR Other Nervous System OR Skin OR B lymphocytes not otherwise specified OR Cytotoxic T cells OR NCI-H23 OR CD4+ T-lymphocytes OR Other NK cells OR Breast Cancer Cell Lines not otherwise specified OR Medulla Oblongata OR Parietal Lobe OR MEF cells OR Crypt OR BT-474 OR Activated Vd1 Gamma-delta T cells OR Activated Vd2 Gamma-delta T cells OR SK-N-SH OR Peripheral blood monocytes OR SNB-75 OR A2780 OR Other Prostate Cancer Cell Lines OR Th2 cells OR Caco2 cells OR EKVX OR MOLT-4 OR Other Pancreatic Cancer Cell Lines OR RXF-393 OR HCT-15 OR Cardiomyocytes OR TK-10 OR RPMI-8266 OR 293 cells OR Lung Cancer Cell Lines not otherwise specified OR Brain OR Heart) AND

(mol. types = biologic drug OR canonical pathway OR chemical - endogenous mammalian OR chemical - endogenous non-mammalian OR chemical - kinase inhibitor OR chemical - other OR chemical - protease inhibitor OR chemical drug OR chemical reagent OR chemical toxicant OR complex OR cytokine OR disease OR enzyme OR function OR fusion gene/product OR G-protein coupled receptor OR group OR growth factor OR ion channel OR kinase OR ligand-dependent nuclear receptor OR mature microRNA OR microRNA OR other OR peptidase OR phosphatase OR transcription regulator OR translation regulator OR transmembrane receptor OR transporter) AND

(data sources = An Open Access Database of Genome-wide Association Results OR BIND OR BioGRID OR Catalogue Of Somatic Mutations In

(c) 2000-2022 QIAGEN. All rights reserved.

Cancer (COSMIC) OR Chemical Carcinogenesis Research Information System (CCRIS) OR Clinical Genome Resource (ClinGen) OR ClinicalTrials.gov OR ClinVar OR Cognia OR DIP OR DrugBank OR Gene Ontology (GO) OR GVK Biosciences OR Hazardous Substances Data Bank (HSDB) OR HumanCyc OR Ingenuity Expert Findings OR Ingenuity ExpertAssist Findings OR IntAct OR Interactome studies OR MIPS OR miRBase OR miRecords OR Mouse Genome Database (MGD) OR Obesity Gene Map Database OR Online Mendelian Inheritance in Man (OMIM) OR TarBase OR TargetScan Human)

Top Canonical Pathways

Name	p-value	Overlap
**Superpathway of Cholesterol Biosynthesis**	5.21E-07	46.4 % 13/28
**Cholesterol Biosynthesis I**	5.81E-06	61.5 % 8/13
**Cholesterol Biosynthesis II (via 24,25-dihydrolanosterol)**	5.81E-06	61.5 % 8/13
**Cholesterol Biosynthesis III (via Desmosterol)**	5.81E-06	61.5 % 8/13
**Valine Degradation I**	8.36E-06	47.6 % 10/21

Top Upstream Regulators

Upstream Regulators

Name	p-value	Predicted Activation
**MAP2K5**	3.21E-06	Activated
**ASPSCR1-TFE3**	2.84E-05	Activated
**SLC29A1**	9.99E-05	Activated

(c) 2000-2022 QIAGEN. All rights reserved.

INGENUITY PATHWAY ANALYSIS

**PTPRR**	2.18E-04	Inhibited
**GABARAPL1**	3.12E-04	Activated

Causal Network

Name	p-value	Predicted Activation
**MAP2K5**	3.21E-06	Activated
**ASPSCR1-TFE3**	2.84E-05	Activated
**SLC29A1**	9.99E-05	Activated
**PTPRR**	2.18E-04	Inhibited
**GABARAPL1**	3.12E-04	Activated

Top Diseases and Bio Functions

Diseases and Disorders

Name	p-value range	# Molecules
**Cancer**	9.16E-03 - 5.33E-62	2001
**Organismal Injury and Abnormalities**	9.16E-03 - 5.33E-62	2008
**Gastrointestinal Disease**	7.76E-03 - 2.46E-43	1791
**Endocrine System Disorders**	9.09E-03 - 2.67E-40	1732
**Neurological Disease**	8.91E-03 - 1.13E-27	1421

Molecular and Cellular Functions

(c) 2000-2022 QIAGEN. All rights reserved.

INGENUITY PATHWAY ANALYSIS

Name	p-value range	# Molecules
**Carbohydrate Metabolism**	7.14E-03 - 1.44E-08	123
**Lipid Metabolism**	9.18E-03 - 8.92E-07	161
**Small Molecule Biochemistry**	9.18E-03 - 8.92E-07	235
**Vitamin and Mineral Metabolism**	9.08E-03 - 8.92E-07	59
**Amino Acid Metabolism**	2.27E-03 - 7.35E-05	34

Physiological System Development and Function

Name	p-value range	# Molecules
**Hematological System Development and Function**	8.79E-04 - 8.79E-04	3
**Nervous System Development and Function**	1.08E-03 - 1.08E-03	4
**Renal and Urological System Development and Function**	1.14E-03 - 1.14E-03	9
**Hair and Skin Development and Function**	1.98E-03 - 1.98E-03	10
**Embryonic Development**	3.26E-03 - 2.44E-03	12

Top Tox Functions

Assays: Clinical Chemistry and Hematology

Name	p-value range	# Molecules
**Increased Levels of ALT**	9.58E-02 - 9.58E-02	1
**Decreased Levels of Albumin**	1.82E-01 - 1.82E-01	1

(c) 2000-2022 QIAGEN. All rights reserved.

**Increased Levels of Alkaline Phosphatase**	3.46E-01 - 3.46E-01	4
**Increased Levels of LDH**	5.06E-01 - 5.06E-01	1

Cardiotoxicity

Name	p-value range	# Molecules
**Cardiac Dysfunction**	1.00E00 - 1.14E-03	18
**Heart Failure**	1.00E00 - 1.14E-03	30
**Cardiac Infarction**	1.00E00 - 2.17E-02	24
**Cardiac Arrythmia**	1.00E00 - 3.32E-02	29
**Tachycardia**	1.00E00 - 3.32E-02	7

Hepatotoxicity

Name	p-value range	# Molecules
**Liver Hyperplasia/Hyperproliferation**	1.00E00 - 1.08E-07	876
**Liver Cirrhosis**	5.96E-01 - 2.29E-02	35
**Liver Fibrosis**	1.00E00 - 2.29E-02	30
**Liver Steatosis**	1.00E00 - 2.93E-02	30
**Hepatocellular carcinoma**	1.00E00 - 4.61E-02	199

Nephrotoxicity

Name	p-value range	# Molecules

(c) 2000-2022 QIAGEN. All rights reserved.

**Renal Necrosis/Cell Death**	3.32E-01 - 6.62E-03	60
**Glomerular Injury**	1.00E00 - 2.58E-02	19
**Kidney Failure**	5.20E-01 - 6.32E-02	25
**Nephrosis**	1.00E00 - 9.58E-02	15
**Renal Damage**	4.67E-01 - 9.58E-02	12

Top Regulator Effect Networks

ID	Regulators	Disease & Functions	Consistency Score
1	ANLN,AURK,SLC29A1,TFEB,TP53COR1	Autophagy,Cell death of epithelial cell lines (+2 more)	10.436
2	ANLN,AURK,CD3 group,SLC29A1,TP53COR1,TP73	Autophagy,Cell death of CD4+ T-lymphocytes (+8 more)	9.864
3	EGLN,MAP2K5,mir-185,NPPB	Concentration of lipid,Synthesis of carbohydrate (+1 more)	3.479
4	GABARAP,GABARAPL1,GABARAPL2	Proteolysis	.0
5	OGA	Metabolism of membrane lipid derivative	.0

Top Networks

ID	Associated Network Functions	Score
1	Cancer, Connective Tissue Disorders, Organismal Injury and Abnormalities	30
2	Carbohydrate Metabolism, Lipid Metabolism, Small Molecule Biochemistry	30

(c) 2000-2022 QIAGEN. All rights reserved.

INGENUITY PATHWAY ANALYSIS

3	Gene Expression, RNA Post-Transcriptional Modification, DNA Replication, Recombination, and Repair	30
4	Embryonic Development, Organismal Development, Gene Expression	28
5	Cell Signaling, Post-Translational Modification, Lipid Metabolism	26

Top Tox Lists

Name	p-value	Overlap
Cholesterol Biosynthesis	4.10E-06	56.2 % 9/16
Pro-Apoptosis	5.38E-03	23.8 % 10/42
TR/RXR Activation	1.31E-02	17.9 % 15/84
PPAR/RXR Activation	1.58E-02	14.8 % 27/183
p53 Signaling	2.59E-02	16.2 % 16/99

Top My Lists

Top My Pathways

(c) 2000-2022 QIAGEN. All rights reserved.

Top Analysis-Ready Molecules

Expr Fold Change

Molecules	Expr. Value	Chart
**AMOT**	↑	
**DDX4**	↑	
**DKK2**	↑	
**PDE1B**	↑	
**PURG**	↑	
**SNAI3**	↑	
**ST8SIA5**	↑	
**CHST9**	↑ 12.388	
**MAN1C1**	↑ 9.411	
**GALNT5**	↑ 8.538	

Expr Fold Change

(c) 2000-2022 QIAGEN. All rights reserved.

INGENUITY PATHWAY ANALYSIS

Analysis Name: H1299 24H
Analysis Creation Date: 2022-01-12
Build version: exported
Content version: 70750971 (Release Date: 2021-10-22)

Experiment Metadata

Name	Value

Analysis Settings

Reference set: Ingenuity Knowledge Base (Genes + Endogenous Chemicals)
Relationship to include: Direct and Indirect
Includes Endogenous Chemicals
Optional Analyses:

Filter Summary:
Consider only molecules and/or relationships where
(species = Human) AND
(tissues/cell lines = Fibroblasts OR RKO OR Other Ovarian Cancer Cell Lines OR Other Cells OR Other Colon Cancer Cell Lines OR OVCAR-3 OR Striatum OR Min6 OR 3T3-L1 cells OR HOP-62 OR Jurkat OR OVCAR-5 OR Prostate Gland OR WEHI-231 OR Central memory cytotoxic T cells OR Other Smooth muscle cells OR Stromal cells OR Chondrocytes OR Neutrophils OR HS 578T OR CD56dim NK cells OR SF-295 OR Lymphoma Cell Lines not otherwise specified OR Effector memory helper T cells OR Pancreas OR Activated helper T cells OR Memory T

(c) 2000-2022 QIAGEN. All rights reserved.

lymphocytes not otherwise specified OR RAW 264.7 OR Corpus Callosum OR Central memory helper T cells OR Pheochromocytoma cell lines not otherwise specified OR Esophagus OR Other Organ Systems OR Keratinocytes OR Large Intestine OR Other CNS Cell Lines OR Peritoneal macrophages OR Effector memory cytotoxic T cells OR Salivary Gland OR Monocytes not otherwise specified OR HepG2 OR HT29 OR Other Myeloma Cell Lines OR Thalamus OR Stomach OR J774 OR Lymph node OR Mesenchymal stem cells OR Skeletal Muscle OR MDA-MB-361 OR Activated CD56dim NK cells OR Placenta OR Ovary OR MALME-3M OR Neurons not otherwise specified OR Other Bone marrow cells OR Peripheral blood lymphocytes OR UACC-257 OR Other Tissues and Primary Cells OR Other Neurons OR SF-268 OR Smooth muscle cells not otherwise specified OR White Matter OR NCI-H332M OR Other Monocyte-derived dendritic cells OR Cos-7 cells OR U2OS OR Myeloma Cell Lines not otherwise specified OR Dendritic cells not otherwise specified OR HCT-116 OR Intraepithelial T lymphocytes OR OVCAR-8 OR OVCAR-4 OR Amygdala OR Vd2 Gamma-delta T cells OR U251 OR Hematopoietic progenitor cells OR Cells not otherwise specified OR Other Breast Cancer Cell Lines OR NCI-H226 OR Other Dendritic cells OR Plasma cells OR THP-1 OR Forestomach OR Mononuclear leukocytes not otherwise specified OR HuH7 OR Other Memory T lymphocytes OR Purkinje cells OR Activated CD56bright NK cells OR Granule cells OR Cerebral Cortex OR Other Stem cells OR Nervous System not otherwise specified OR BT-549 OR Embryonic stem cells OR A375 OR Stem cells not otherwise specified OR A549-ATCC OR Caudate Nucleus OR Cerebral Ventricles OR HL-60 OR Eosinophils OR HUVEC cells OR Other Mononuclear leukocytes OR HeLa OR NB4 OR Splenocytes OR INS-1 OR Other Epithelial cells OR Cervical cancer cell line not otherwise specified OR Other Macrophage Cancer Cell Lines OR Other B lymphocytes OR Sertoli cells OR Epidermis OR Lung OR Prostate Cancer Cell Lines not otherwise specified OR SN12C OR Kidney cell lines not otherwise specified OR Osteosarcoma Cell Lines not otherwise specified OR T47-D OR H460 OR Other Cell Line OR PC-3 OR Pre-B lymphocytes OR Hypothalamus OR Pituitary Gland OR Vascular smooth muscle cells OR SR OR RBL-2H3 OR Bone marrow-derived macrophages OR Thymocytes OR Other Immune cells OR PBMCs OR Other Lung Cancer Cell Lines OR Thyroid Gland OR J-774A.1 OR BDCA-1+ dendritic cells OR Trigeminal Ganglion OR Other Lymphoma Cell Lines OR SF-539 OR Ovarian Cancer Cell Lines not otherwise specified OR Pyramidal neurons OR SW-480 OR HMC-1 OR PANC-1 OR Substantia Nigra OR U87MG OR CD34+ cells OR Cornea OR Dermis OR LNCaP cells OR SK-MEL-28 OR MDA-N OR Putamen OR Effector T cells OR Kidney Cancer Cell Lines not otherwise specified OR KM-12 OR Granulocytes not otherwise specified OR Murine NKT cells OR UO-31 OR Other Macrophages OR Subventricular Zone OR Spleen OR Mature monocyte-derived dendritic cells OR Myeloid dendritic cells OR Other Melanoma Cell Lines OR Immune cells not otherwise specified OR IGROV1 OR Other Cervical cancer cell line OR Retina OR Osteoblasts OR Nucleus Accumbens OR Other Fibroblast cell lines OR Smooth Muscle OR Plasmacytoid dendritic cells OR K-562 OR Other Leukemia Cell Lines OR Other Teratocarcinoma Cell Lines OR SW-620 OR Endothelial cells not otherwise specified OR Bladder OR Kidney OR Adrenal Gland OR HEL OR Pro-B lymphocytes OR Cortical neurons OR Gray Matter OR Macrophages not otherwise specified OR NCI-ADR-RES OR Teratocarcinoma Cell Lines not otherwise specified OR Dorsal Root Ganglion OR Mast cells OR Naive B cells OR Thymus OR Neuroblastoma Cell Lines not otherwise specified OR Other Osteosarcoma Cell Lines OR Pancreatic Cancer Cell Lines not otherwise specified OR Other Monocytes OR SK-MEL-5 OR

(c) 2000-2022 QIAGEN. All rights reserved.

INGENUITY PATHWAY ANALYSIS

A498 OR Fibroblast cell lines not otherwise specified OR Microglia OR Other Kidney cell lines OR Epithelial cells not otherwise specified OR 786-0 OR Blood platelets OR Small Intestine OR Peripheral blood leukocytes not otherwise specified OR Cerebellum OR Astrocytes OR ACHN OR Testis OR Cell Line not otherwise specified OR U266 OR NCI-H522 OR Trachea OR Swiss 3T3 cells OR Th1 cells OR MDA-MB-435 OR Other Hepatoma Cell Lines OR NIH/3T3 cells OR Other Kidney Cancer Cell Lines OR Th17 cells OR MDA-MB-231 OR Natural T-regulatory cells OR Other Peripheral blood leukocytes OR Other Neuroblastoma Cell Lines OR PC-12 cells OR Other T lymphocytes OR Adipocytes OR Adipose OR HOP-92 OR Hepatoma Cell Lines not otherwise specified OR HCC-2998 OR Bone marrow-derived dendritic cells OR Sciatic Nerve OR Memory B cells OR NK cells not otherwise specified OR Other Pheochromocytoma cell lines OR Calvaria OR CCRF-CEM OR Hippocampus OR Colon Cancer Cell Lines not otherwise specified OR Mammary Gland OR Spinal Cord OR CAKI-1 OR NT2/D1 OR Monocyte-derived macrophage OR BA/F3 OR MCF7 OR Immature monocyte-derived dendritic cells OR Hepatocytes OR Effector memory RA+ cytotoxic T cells OR UACC-62 OR Organ Systems not otherwise specified OR Other Granulocytes OR Ventricular Zone OR Leukemia Cell Lines not otherwise specified OR CNS Cell Lines not otherwise specified OR Liver OR Vd1 Gamma-delta T cells OR Beta islet cells OR Lens OR LOX IMVI OR M14 OR Melanocytes OR Bone marrow cells not otherwise specified OR DU-145 OR U937 OR Monocyte-derived dendritic cells not otherwise specified OR Langerhans cells OR Megakaryocytes OR Uterus OR Granulosa cells OR COLO205 OR Immune cell lines not otherwise specified OR CD56bright NK cells OR Lymphocytes not otherwise specified OR Melanoma Cell Lines not otherwise specified OR Brainstem OR Microvascular endothelial cells OR Choroid Plexus OR Granule Cell Layer OR Other Immune cell lines OR Hep3B OR BDCA-3+ dendritic cells OR T lymphocytes not otherwise specified OR Olfactory Bulb OR Tissues and Primary Cells not otherwise specified OR SK-OV-3 OR Other Lymphocytes OR SK-MEL-2 OR Other Endothelial cells OR Oocytes OR Naive helper T cells OR MG-63 OR Cartilage Tissue OR Macrophage Cancer Cell Lines not otherwise specified OR P19 OR MDA-MB-468 OR Other Nervous System OR Skin OR B lymphocytes not otherwise specified OR Cytotoxic T cells OR NCI-H23 OR CD4+ T-lymphocytes OR Other NK cells OR Breast Cancer Cell Lines not otherwise specified OR Medulla Oblongata OR Parietal Lobe OR MEF cells OR Crypt OR BT-474 OR Activated Vd1 Gamma-delta T cells OR Activated Vd2 Gamma-delta T cells OR SK-N-SH OR Peripheral blood monocytes OR SNB-75 OR A2780 OR Other Prostate Cancer Cell Lines OR Th2 cells OR Caco2 cells OR EKVX OR MOLT-4 OR Other Pancreatic Cancer Cell Lines OR RXF-393 OR HCT-15 OR Cardiomyocytes OR TK-10 OR RPMI-8266 OR 293 cells OR Lung Cancer Cell Lines not otherwise specified OR Brain OR Heart) AND

(mol. types = biologic drug OR canonical pathway OR chemical - endogenous mammalian OR chemical - endogenous non-mammalian OR chemical - kinase inhibitor OR chemical - other OR chemical - protease inhibitor OR chemical drug OR chemical reagent OR chemical toxicant OR complex OR cytokine OR disease OR enzyme OR function OR fusion gene/product OR G-protein coupled receptor OR group OR growth factor OR ion channel OR kinase OR ligand-dependent nuclear receptor OR mature microRNA OR microRNA OR other OR peptidase OR phosphatase OR transcription regulator OR translation regulator OR transmembrane receptor OR transporter) AND

(data sources = An Open Access Database of Genome-wide Association Results OR BIND OR BioGRID OR Catalogue Of Somatic Mutations In

(c) 2000-2022 QIAGEN. All rights reserved.

Cancer (COSMIC) OR Chemical Carcinogenesis Research Information System (CCRIS) OR Clinical Genome Resource (ClinGen) OR ClinicalTrials.gov OR ClinVar OR Cognia OR DIP OR DrugBank OR Gene Ontology (GO) OR GVK Biosciences OR Hazardous Substances Data Bank (HSDB) OR HumanCyc OR Ingenuity Expert Findings OR Ingenuity ExpertAssist Findings OR IntAct OR Interactome studies OR MIPS OR miRBase OR miRecords OR Mouse Genome Database (MGD) OR Obesity Gene Map Database OR Online Mendelian Inheritance in Man (OMIM) OR TarBase OR TargetScan Human)

Top Canonical Pathways

Name	p-value	Overlap
**Melatonin Signaling**	1.95E-06	32.9 % 23/70
**CLEAR Signaling Pathway**	3.01E-05	19.9 % 56/281
**Superpathway of Cholesterol Biosynthesis**	1.56E-04	39.3 % 11/28
**Cholesterol Biosynthesis I**	2.47E-04	53.8 % 7/13
**Cholesterol Biosynthesis II (via 24,25-dihydrolanosterol)**	2.47E-04	53.8 % 7/13

Top Upstream Regulators

Upstream Regulators

Name	p-value	Predicted Activation
**MAP2K5**	1.29E-05	Activated
**GSTO1**	1.44E-04	Inhibited
**PTPRR**	3.99E-04	Inhibited
**MGAT1**	7.78E-04	

(c) 2000-2022 QIAGEN. All rights reserved.

CHCHD5	9.90E-04	Activated

Causal Network

Name	p-value	Predicted Activation
MAP2K5	1.29E-05	Activated
GSTO1	1.44E-04	Inhibited
PTPRR	3.99E-04	Inhibited
MGAT1	7.78E-04	
RFX1	9.17E-04	

Top Diseases and Bio Functions

Diseases and Disorders

Name	p-value range	# Molecules
Cancer	4.63E-03 - 1.65E-89	2417
Organismal Injury and Abnormalities	4.67E-03 - 1.65E-89	2426
Gastrointestinal Disease	4.65E-03 - 1.46E-82	2207
Endocrine System Disorders	4.65E-03 - 6.71E-79	2153
Neurological Disease	4.42E-03 - 3.49E-55	1750

Molecular and Cellular Functions

(c) 2000-2022 QIAGEN. All rights reserved.

INGENUITY PATHWAY ANALYSIS

Name	p-value range	# Molecules
Cellular Assembly and Organization	3.82E-03 - 2.48E-09	229
Cellular Function and Maintenance	3.82E-03 - 2.48E-09	282
Carbohydrate Metabolism	4.67E-03 - 1.31E-07	150
Cell Morphology	3.97E-03 - 1.35E-07	202
Molecular Transport	4.67E-03 - 4.52E-07	242

Physiological System Development and Function

Name	p-value range	# Molecules
Embryonic Development	2.42E-03 - 1.77E-04	14
Organismal Development	3.34E-03 - 1.77E-04	50
Tissue Development	1.77E-04 - 1.77E-04	4
Renal and Urological System Development and Function	3.97E-03 - 1.04E-03	20
Hematological System Development and Function	1.54E-03 - 1.54E-03	3

Top Tox Functions

Assays: Clinical Chemistry and Hematology

Name	p-value range	# Molecules
Increased Levels of ALT	1.15E-01 - 1.15E-01	1
Decreased Levels of Albumin	2.18E-01 - 2.18E-01	1

(c) 2000-2022 QIAGEN. All rights reserved.

INGENUITY PATHWAY ANALYSIS

Increased Levels of CRP	2.18E-01 - 2.18E-01	1
Increased Levels of Albumin	3.08E-01 - 3.08E-01	1
Increased Levels of Alkaline Phosphatase	4.88E-01 - 4.88E-01	4

Cardiotoxicity

Name	p-value range	# Molecules
Cardiac Dysfunction	1.00E00 - 4.16E-03	27
Heart Failure	1.00E00 - 4.16E-03	34
Cardiac Dilation	1.00E00 - 2.16E-02	37
Cardiac Enlargement	1.00E00 - 2.16E-02	42
Cardiac Arrythmia	1.00E00 - 2.27E-02	40

Hepatotoxicity

Name	p-value range	# Molecules
Liver Hyperplasia/Hyperproliferation	1.00E00 - 4.46E-19	1133
Hepatocellular carcinoma	1.00E00 - 1.16E-02	254
Liver Steatosis	1.00E00 - 6.82E-02	28
Hepatocellular Peroxisome Proliferation	6.25E-01 - 1.15E-01	3
Liver Cholestasis	5.76E-01 - 1.15E-01	9

Nephrotoxicity

(c) 2000-2022 QIAGEN. All rights reserved.

INGENUITY PATHWAY ANALYSIS

Name	p-value range	# Molecules
**Nephrosis**	1.00E00 - 1.37E-03	23
**Glomerular Injury**	1.00E00 - 3.69E-02	19
**Renal Dysplasia**	1.93E-01 - 3.69E-02	4
**Renal Necrosis/Cell Death**	1.00E00 - 4.64E-02	62
**Kidney Failure**	1.00E00 - 1.15E-01	23

Top Regulator Effect Networks

ID	Regulators	Disease & Functions	Consistency Score
**1**	CD3 group,CHCHD5,MAP2K5,MIEF2,mir-185,NPPB	Concentration of lipid (+3 more)	5.019
**2**	Interferon alpha	I-kappaB kinase/NF-kappaB cascade	-7.0
**3**	OGA	Concentration of lipid	-9.827

Top Networks

ID	Associated Network Functions	Score
**1**	Dermatological Diseases and Conditions, Immunological Disease, Inflammatory Disease	28
**2**	Lipid Metabolism, Small Molecule Biochemistry, Vitamin and Mineral Metabolism	28

(c) 2000-2022 QIAGEN. All rights reserved.

INGENUITY PATHWAY ANALYSIS

**3**	Cell Cycle, Cardiovascular System Development and Function, Cellular Development	26
**4**	Inflammatory Response, DNA Replication, Recombination, and Repair, Gene Expression	26
**5**	Cell Cycle, DNA Replication, Recombination, and Repair, Cellular Assembly and Organization	26

Top Tox Lists

Name	p-value	Overlap
**Cholesterol Biosynthesis**	1.71E-04	50.0 % 8/16
**Glutathione Depletion - CYP Induction and Reactive Metabolites**	8.05E-03	41.7 % 5/12
**Hormone Receptor Regulated Cholesterol Metabolism**	8.45E-03	50.0 % 4/8
**PPAR/RXR Activation**	3.03E-02	16.4 % 30/183
**Fatty Acid Metabolism**	3.08E-02	18.4 % 18/98

Top My Lists

(c) 2000-2022 QIAGEN. All rights reserved.

INGENUITY PATHWAY ANALYSIS

Top My Pathways

Top Analysis-Ready Molecules

Expr Fold Change

Molecules	Expr. Value	Chart
AGT	↑	
ANKRD60	↑	
APOBEC3B	↑	
CFAP157	↑	
CLUL1	↑	
CYP2W1	↑	
DRC1	↑	
FOXQ1	↑	
GPRIN2	↑	
GREM2	↑	

Expr Fold Change

(c) 2000-2022 QIAGEN. All rights reserved.

INGENUITY PATHWAY ANALYSIS
